# The use of cannabis for medical reasons in the UK: Prescriptions, sources, products, and high-risk use

**DOI:** 10.1017/S0033291726105327

**Published:** 2026-07-31

**Authors:** Elle Wadsworth, David Hammond, Tom P. Freeman

**Affiliations:** 1 Centre for Drug Use Research, Department of Psychology, University of Bath, Bath, UK; 2 School of Public Health Sciences, University of Waterloo, Waterloo, Canada

**Keywords:** access, cannabis, cannabis sources, CUDITmedical cannabis, potency

## Abstract

**Background:**

The United Kingdom legalized medical cannabis in 2018, yet little is known about people using cannabis medically with and without a prescription. This study aimed to estimate: 1) the percentage of people reporting using cannabis medically with and without a prescription; 2) the sources; 3) products used; and 4) associations between frequent product use and medical cannabis status.

**Methods:**

Data were from national repeat cross-sectional surveys conducted in 2023 and 2024. UK participants were aged 16–65 who used cannabis in the past 12 months (*n* = 4,414). Multivariable regression analyses estimated associations between outcomes and medical cannabis status.

**Results:**

Overall, 12.9% of respondents reported receiving a medical cannabis prescription, 35.9% reported medical use without a prescription, and 51.2% reported no medical use. Cannabis was sourced through diverse routes; only 10.9% of people with prescriptions obtained all their cannabis from a prescription. Respondents with a prescription had a higher probability of reporting frequent use of drops, capsules, vape oils, edibles, drinks, solid concentrates, hash/kief, and topicals (aRRs = range between 1.82 and 3.51)) and a higher probability of screening positive for high-risk use (aRR = 3.09; 2.51–3.79) than respondents who did not use medically.

**Conclusions:**

People with medical cannabis prescriptions show a higher probability of frequent use of processed products and of meeting a threshold for high-risk use. Healthcare encounters during medical cannabis prescribing should discuss risks related to products, potency, and adverse effects such as cannabis use disorder.

## Background

Cannabis is the most used illegal drug in the United Kingdom, with 7.6% and 6.8% of adults in England and Wales reporting past-year use in 2023 and 2024 (Office for National Statistics, 2024). In addition to using cannabis for nonmedical (or ‘recreational’) use, cannabis can be consumed for therapeutic benefits. Evidence for the effectiveness of cannabis for physical and mental health conditions is limited (Freeman, Morgan, & Hindocha, 2019; Hsu et al., 2026; National Institute for Health and Care Excellence, 2019). Regardless, cannabis is still widely used for potential therapeutic benefits such as pain relief (Kosiba, Maisto, & Ditre, 2019; Walsh et al., 2013), with stronger evidence of effectiveness pointing to neuropathic pain, cancer pain, chemotherapy-induced nausea, and multiple sclerosis spasticity symptoms (National Academies of Sciences, 2017). Cross-sectional surveys among UK adults show that consumers reporting using cannabis for medical reasons were most likely to report using cannabis to manage depression, anxiety, and chronic pain (Couch, 2020; Erridge, Coomber, & Sodergren, 2022; Erridge, Troup, & Sodergren, 2024). Yet systematic reviews and meta-analyses have found no evidence for cannabinoids in treating anxiety and an absence of evidence for depression (Wilson et al., 2026).

In recent years, medical and nonmedical cannabis policies have become more permissive globally (Freeman et al., 2026). In the United Kingdom, nonmedical cannabis is illegal; however, unapproved/unlicensed cannabis-based products for medical use (CBPM) became legal to prescribe in November 2018. Prescriptions for CBPMs can be obtained from doctors on the specialist register of the General Medical Council, either through the UK’s universal healthcare system, the National Health Service (NHS), or through private, for-profit cannabis clinics (Arjun et al., 2025). These prescriptions are intended for those who have exhausted other recommended treatment options. Access to prescriptions for CBPMs given through the NHS remains very limited, with the majority accessing through private clinics (UK Parliament, 2023). Limited prescribing may reflect insufficient evidence of efficacy and safety (NHS England, 2023). Prescribing an unlicensed product requires the prescriber to accept increased medico-legal responsibilities, which some may be unwilling to undertake (NHS England, 2023). Moreover, prescribers may be cautious about substances with dependence potential, given historical experiences with drugs such as benzodiazepines, which were widely prescribed before their addictive risks were fully recognized (Lader, 2011). This legacy may contribute to a more conservative approach among doctors when considering CBPMs prescriptions. In contrast to the small number of consumers accessing CBPMs legally, the use of ‘illegal’ cannabis for medical purposes is widespread (Couch, 2020; Erridge et al., 2024). A nationally representative survey estimated that more than one million UK residents consumed cannabis for medical purposes (Erridge et al., 2024). Thus, there appears to be an unmet demand for medical cannabis among consumers not accessing it legally – perhaps due to limited evidence of efficacy and safety, medical need as assessed by a healthcare professional, affordability, accessibility, or stigma (Case, 2020; Heeg, Morari, Lynskey, & Turner, 2024; Wilson & McGrath, 2023). Beyond patient outcome data from medical cannabis registries, little is known about those accessing cannabis legally via prescription, or those using cannabis for medical purposes but sourcing it illegally, including the types of products consumed.

Cannabis contains over 100 cannabinoids, and the primary cannabinoids of interest are Δ9-tetrahydrocannabinol (THC), which is the main psychoactive compound, and cannabidiol (CBD), a compound that is not intoxicating when used alone. The THC concentration of dried flower has increased over several decades (Freeman et al., 2021). Rising potency is a public health concern due to associations with high-potency products and elevated risk of psychotic disorders and cannabis use disorders (Freeman & Winstock, 2015; Hall & Degenhardt, 2015; Petrilli et al., 2022). The psychoactive effects of cannabis depend on the type of product (e.g. dried flower or ‘processed’ products such as extracts), route of administration (e.g. smoking), and potency. Smoking dried flower with tobacco remains the most common method of cannabis consumption in the United Kingdom; however, evidence suggests diversification toward processed (i.e. non-flower) products, which can enable cannabinoid concentrations higher than those naturally synthesized in the plant (Hammond, Wadsworth, Reid, & Burkhalter, 2021). It is unclear whether the patterns in the broader UK cannabis market are mirrored among medical cannabis users, or what the implications of such product use may be. Not all product forms are permitted in the legal medical market. Permitted forms of unapproved/unlicensed CBPMs in the UK medical market include dried flower (recommended for vaping), oils/tinctures, capsules, vape cartridges, and some edibles (e.g. pastilles). Other processed products, such as hash and solid concentrates, are not permitted (Medicines and Healthcare Products Regulatory Agency, 2014; National Institute for Health and Care Excellence, 2019). However, the level of adherence to this guidance is unclear. It is therefore critical to assess the type of products consumed by people using cannabis for medical reasons, to understand health effects in this important subgroup, which has not been characterized in previous studies.

## Objective

The UK Advisory Council for the Misuse of Drugs (ACMD) assessed the impact of rescheduling medical cannabis in 2020 and stated that more data and research were needed (Advisory Council on the Misuse of Drugs, 2020). In 2025, they issued a call for evidence into CBPMs, requesting information on barriers to access, perceptions, and unintended consequences of the policy change (Advisory Council on the Misuse of Drugs, 2025). Since legalization in 2018, many data gaps remain that must be addressed for the ACMD to fully assess the effectiveness of medical cannabis provision in the United Kingdom and make recommendations moving forward. Accordingly, the aims of the study were to estimate: 1) the percentage of people who use cannabis for medical purposes and who have and do not have a prescription for CBPMs (hereafter: ‘medical cannabis prescription’); 2) the sources used to obtain cannabis; 3) the products used; and 4) associations between product use and receiving a medical cannabis prescription.

## Methods

Data were drawn from the 2023 and 2024 waves of the International Cannabis Policy Study (ICPS), a series of repeat cross-sectional surveys conducted annually in Canada, the United States, Australia, New Zealand, the United Kingdom, and Germany. The current study analyzed data from the UK sample only. Data were collected through self-completed web-based surveys conducted between September and November in 2023 and 2024. Non-probability samples of respondents aged 16–65 were recruited through the Nielsen Consumer Insights Global Panel and its partners’ panels. Nielsen selects stratified random samples from the online panels, with quotas based on sex and age. For the UK sample, people who had used cannabis in the past 12 months were oversampled to ensure sufficient power for analyses. Respondents were not resampled. Upon completion, respondents received remuneration in accordance with their panel’s usual incentive structure. The American Association for Public Opinion Research cooperation rate (American Association for Public Opinion Research, 2016), which is the percentage of respondents who completed the survey among all eligible respondents who accessed the survey link, was 55.1% in 2023 and 39.1% in 2024. The median survey time was 22 minutes.

The study was reviewed by and received ethics clearance through the University of Waterloo (ORE#31330) and the University of Bath (0513–586). Informed consent was obtained from all respondents and/or their legal guardian(s). A full description of the study methods, including sampling and post-stratification weighting, can be found in the ICPS Technical Reports (Fataar et al., 2025; Iraniparast et al., 2023). The analysis plan for this paper was pre-registered on the Open Science Framework prior to data analysis (https://osf.io/j7ax6).

### Measures

#### Socio-demographic measures

Sex-at-birth, age, ethnicity/race, highest education level, perceived income adequacy, and region (Table 1). For ‘perceived income adequacy’ and ‘ethnicity/race,’ those who answered ‘Don’t know’ or ‘Refuse to answer’ were categorized as ‘Unstated’. The capital city (London) was separated from all other regions within England.

**Table 1.** Unweighted and weighted sample characteristics among UK respondents who reported consuming cannabis in the past year in 2023–2024 (*n* = 4,415) Table 1. long description.

	Unweighted% (*n*)	Weighted% (*n*)
**Year**		
2023	36.0% (1590)	50.6% (2235)
2024	64.0% (2825)	49.4% (2180)
**Age**		
16–25	22.3% (983)	37.6% (1659)
26–35	30.0% (1324)	23.7% (1046)
36–45	28.9% (1275)	22.1% (977)
46–55	10.8% (475)	9.3% (410)
56–65	8.1% (358)	7.3% (321)
**Sex at birth**		
Female	45.4% (2005)	39.7% (1753)
Male	54.6% (2410)	60.3% (2662)
**Race/ethnicity**		
Asian or Asian British	5.5% (242)	5.5% (245)
Black, Black British, Caribbean or African	9.8% (434)	7.6% (336)
Mixed or multiple ethnic groups	5.2% (229)	5.1% (226)
White	78.3% (3455)	80.3% (3546)
Other/unstated	1.3% (55)	1.4% (62)
**Highest level of education**		
Less than high school	11.1% (492)	19.2% (848)
High school diploma	13.9% (614)	17.6% (776)
Some college or technical vocation	34.1% (1505)	34.0% (1502)
Bachelor’s degree or higher	38.8% (1714)	26.8% (1185)
Unstated	2.0% (90)	2.4% (104)
**Income adequacy**		
Very difficult or difficult	24.8% (1095)	26.6% (1175)
Neither easy nor difficult	31.3% (1383)	32.9% (1454)
Very Easy or easy	41.5% (1834)	37.9% (1673)
Unstated	2.3% (103)	2.6% (113)
**Region**		
England not including London	61.0% (2691)	59.8% (2639)
London	26.3% (1163)	21.8% (963)
Wales	4.0% (175)	3.9% (173)
Scotland	7.2% (318)	11.8% (521)
Northern Ireland	1.5% (68)	2.7% (119)
**Cannabis use frequency**		
Less than monthly, but in the past year	27.8% (1226)	28.0% (1236)
Monthly	25.3% (1117)	24.7% (1092)
Weekly	19.8% (874)	18.8% (832)
Daily or near daily	27.1% (1198)	28.4% (1255)
**Medical cannabis status**		
People with a prescription	15.3% (676)	12.9% (570)
People without a prescription that use medically	35.2% (1554)	35.9% (1584)
No reported medical use	48.1% (2125)	51.2% (2261)

#### High-risk use using the CUDIT-SF

High-risk use was assessed using the Cannabis Use Disorder Identification Test Revised Short Form (CUDIT-SF) (Bonn-Miller et al., 2016). The CUDIT-SF is a three-item form that determines a positive screen to be two or above, which was found to correctly identify 78.26% of people with DSM-5 cannabis use disorder in a sample recruited from a medical cannabis dispensary (AUC = 0.84) and 78.31% in an independent sample reporting recreational use (AUC = 0.85) (Bonn-Miller et al., 2016).

#### Medical cannabis use status

Medical cannabis use status was derived from two questions. First, ‘Do you use cannabis for medical reasons, recreational reasons, or both? By medical cannabis user, we mean someone who uses cannabis only to manage a medical condition’. (1 = Medical use only, 2 = Recreational use only, 3 = Both recreational and medical use, 4 = Do not know). Second, ‘Did you receive a prescription to use medical cannabis at any time in the past 12 months?’ (5 = Yes, 6 = No, 7 = Do not know). Responses to these questions were combined to create three exclusive categories: ‘People with a medical cannabis prescription’ [5], ‘People without a medical cannabis prescription who use medically’ [1,3,6,7], ‘No medical use’ [2,4].

#### Asked for a medical cannabis prescription

Respondents were asked, ‘Have you ever asked a licensed health professional for a prescription to use medical cannabis?’ with response options ‘Yes’ and ‘No’ (No/Do not know).

#### Cannabis product use and frequency

Respondents were asked whether they had used any of the nine product types in the past 12 months: dried flower, oils or liquids taken orally (drops or capsules, oil or liquid for vaping, edibles/foods, drinks, solid concentrates, hash/kief, tinctures, and topicals. Respondents who reported using a product were asked to indicate their frequency of use for each product. Drops and capsules were combined for analyses of past 12-month use but were separated for frequency of use, in accordance with the survey design. Responses were dichotomized to a ‘Monthly or more frequent use (MMF)’ (Monthly/Weekly/Daily) vs ‘Other’ (Less than monthly/Do not know/Not applicable – i.e. those who did not use the product but used cannabis in the past 12 months). Tinctures were not included in the analysis due to low cell counts and the resulting inability of models to converge.

#### Cannabis product sources

Respondents could select all that applied to the following question: ‘In the past 12 months, have you gotten any type of cannabis from the following sources?’ (I made or grew my own/From a family member or friend/From a dealer/Online/From a store or dispensary/Through medical prescription/Other). All were binary questions whereby selecting the source was ‘Yes’, and the absence was ‘No’. ‘Other’ was recategorized to existing options if provided in open text.

#### Source of medical cannabis prescription

Respondents who had reported obtaining cannabis through a medical prescription were asked to indicate the source, with options including NHS prescription, private prescription, or patient registry.

#### Legally sourced medical cannabis

Respondents were asked ‘Overall, how much of the cannabis that you used in the past 12 months was from a legal medical prescription?’ Respondents were categorized into four options ‘All’ (100%), ‘Some’ (1-99%), ‘None’ (0%), and ‘Unstated’ (I did not buy or pay for cannabis in the past 12 months/Do not know/Refuse to answer).

All questions included ‘Don’t know’ and ‘Refuse to answer’ response options, which were excluded from analyses unless otherwise specified in the measures above. It should be noted that the ICPS survey distinguishes between questions on THC-containing cannabis products and CBD-only products. The questions analyzed in this study did not include responses for CBD-only products.

### Analysis

The current analysis draws on pooled UK data from 2023 (*n* = 4,010) and 2024 (*n* = 7,002). After removing respondents due to self-reported dishonesty determined by asking participants, ‘Were you able to provide “honest” answers about your cannabis use during the survey?’ (*n* = 256); poor data quality determined by those who did not select the current month (*n* = 813); those who identified as intersex and an ‘other’/unstated sex due to insufficient cell counts for weighting (*n* = 10); speeding (*n* = 66); duplicate entries (*n* = 272); and unstated region (*n* = 22), 9,573 respondents were retained. The pooled analytic sample for the current study was 4,415 respondents, which includes data from people who reported using cannabis in the past 12 months (*n*_2023_ = 1,590; *n*_2024_ = 2,825). Missing data were removed using case-wise deletion for variables used in regression models: education (*n* = 201; 2.1% of the analytic sample), asking a licensed healthcare professional for a prescription (*n* = 83; 1.9%), and medical cannabis status use (*n* = 60; 1.4%).

Post-stratification sample weights were constructed to calibrate to population proportions as available in censuses and national benchmark surveys. Respondents were classified into age-by-sex-by-region groups, ethnicity-by-region groups, education groups, and age-by-sex-by-cannabis-use groups. Correspondingly grouped population proportion estimates were obtained from national government agencies (Fataar et al., 2025; Iraniparast et al., 2023). A ranking algorithm was applied to compute weights that are calibrated to these groupings. The SAS macro ‘RAKE_AND_TRIM_G4_V5’ was used, with trimming to 5 (rescaled) if necessary. Estimates are weighted unless otherwise specified.

First, descriptive statistics were used to estimate the percentage of 1) people using cannabis for medical purposes with and without a prescription; 2) the use of cannabis products; and 3) the sources used to obtain cannabis – by medical cannabis use status. Second, Rao-Scott chi-square tests of association were used between medical cannabis use status for source and product variables. Third, univariable and multivariable regression models were used to test the association between MMF product use and medical cannabis use status. Post-hoc sensitivity analyses that were not pre-registered were conducted where any MMF use (vs no MMF use) of all nine products was the outcome and where the proportion of cannabis used from a legal prescription was the predictor instead of receiving a prescription.

Three additional post-hoc analyses that were not pre-registered were conducted on 1) the percentage of perceived ease of accessing legal medical cannabis, including Rao-Scott chi-square tests of association between medical cannabis use status; 2) multivariable regression models to test the association between asking for a medical cannabis prescription; and 3) receiving a medical cannabis prescription, high-risk use, and socio-demographic characteristics.

All models were adjusted for region, age, sex-at-birth, education, ethnicity/race, income adequacy, year, and device used to complete the survey. Adjusted risk ratios (aRR) are reported with 95% confidence intervals (95% CI). We used quasi-Poisson regression analyses with a log-link function to obtain adjusted risk ratios (Norton, Dowd, & Maciejewski, 2018). Analyses were conducted using R version 4.5.1, with survey weighting and svyglm for models.

## Findings

Table 1 displays the sample characteristics of respondents who reported using cannabis in the past year. The sample was predominantly aged between 16 and 35 (61.3%), male-at-birth (60.3%), identified as White race/ethnicity (80.3%), and educated beyond high school (60.8%). Close to a quarter of people reported daily cannabis use (28.4%). A total of 12.9% reported receiving a medical cannabis prescription in the past year, 35.9% reported use of cannabis medically but without a medical cannabis prescription, and 51.2% did not report medical cannabis use.

### Medical cannabis sources

A total of 18.5% of people who used cannabis reported ever asking for a medical cannabis prescription (Table 2). A total of 7.2% reported getting their cannabis from a medical prescription in the past year. When disaggregated by medical cannabis use status, 29.1% of people who reported receiving a prescription, 7.0% of people without a prescription who reported using medically, and 2.1% of people who did not report medical use reported getting cannabis from a prescription (*χ* ^2^ = 486.4, *p* < 0.001). Small percentages of people who used cannabis reported getting their prescription from the NHS (2.7%) and privately (4.0%). Among people who reported receiving a medical cannabis prescription, 2.9%, 62.9%, and 10.9% reported that none, some, and all of their cannabis was from a legal medical prescription, respectively. Greater percentages of people who reported receiving a prescription screened positive for high-risk use (75.8%) compared to those without a prescription who reported using medically (45.5%), or those who reported no medical use (39.6%; *χ* ^2^ = 240.1, *p* < 0.001).

**Table 2.** Percentages of cannabis outcomes among people with and without a prescription to use medical cannabis and those who did not report medical use, 2023–2024 (*n* = 4,415) Table 2. long description.

	People who reported using cannabis in the past year (*n* = 4,415)	People with a medical cannabis prescription in past year (*n* = 676)	People without a prescription that use medically (*n* = 1,554)	People who did not report medical use (*n* = 2,125)	*χ* ^2^, *p*-value
	**Weighted % (unweighted n)**				
**Ever asked for a medical cannabis prescription**					
Yes	18.5% (931)	83.2% (570)	13.1% (228)	6.1% (132)	1841.2, *p* < 0.001
**Sourced cannabis from medical prescription in past year**					
Yes	7.2% (370)	29.1% (201)	7.0% (112)	2.1% (56)	486.4, *p* < 0.001
**Source of medical prescription**					
Through an NHS prescription	2.7% (132)	11.6% (78)	2.7% (39)	0.5% (15)	509.5, *p* < 0.001
Through a private prescription	4.0% (211)	16.7% (114)	3.5% (62)	1.3% (24)	
Through a patient registry	0.4% (21)	0.8% (9)	0.5% (7)	0.3% (5)	
**How much of the cannabis used in the past year was from a legal medical prescription**					
None (0%)	35.5% (1492)	2.9% (15)	41.0% (585)	40.5% (886)	984.8, *p* < 0.001
Some (1–99%)	21.1% (1169)	62.9% (468)	19.4% (385)	12.0% (306)	
All (100%)	3.0% (144)	10.9% (67)	2.8% (39)	0.9% (32)	
Unstated	40.5% (1610)	23.3% (126)	36.9% (545)	46.5% (901)	
**Sources of cannabis used in past year**					
From a dealer (in person)	51.9% (2321)	47.2% (352)	54.1% (836)	52.5% (1114)	8.1, *p* = 0.094
From a family member or friend	43.7% (1960)	32.6% (233)	44.3% (683)	46.4% (1026)	35.3, *p* < 0.001
From a store or dispensary	15.4% (792)	36.3% (271)	14.7% (243)	10.8% (269)	227.7, *p* < 0.001
Internet delivery service or mailed to me	13.9% (713)	28.5% (219)	14.8% (261)	9.7% (227)	135.6, *p* < 0.001
I made or grew my own	8.9% (443)	32.0% (200)	7.7% (135)	4.0% (102)	446.2, *p* < 0.001
**High-risk use from CUDIT-SF score**					
Positive screen	45.9% (2002)	75.8% (497)	45.5% (688)	39.6% (807)	240.1, *p* < 0.001
Negative screen	54.1% (2413)	24.2% (179)	54.5% (866)	60.4% (1318)	

In the past year, people who used cannabis reported getting cannabis from a dealer (51.9%), a family member or friend (43.7%), a physical store (15.4%), online (13.9%), and by making or growing their own (8.9%). Greater percentages of people who reported receiving a prescription reported making or growing their own cannabis (*χ* ^2^ = 446.2, *p* < 0.001), getting their cannabis online (*χ* ^2^ = 135.6, *p* < 0.001), and from a physical store (*χ* ^2^ = 227.7, *p* < 0.001). Greater percentages of people without a prescription – both those who reported using medically and those who did not – reported getting their cannabis from a family member or friend (*χ* ^2^ = 35.3, *p* < 0.001). There was no difference between medical cannabis use status for getting cannabis from a dealer (*χ* ^2^ = 8.1, *p* = 0.094).

### Perceived access to medical cannabis

Two-thirds of people who reported receiving a prescription reported perceiving access to a prescription as easy (66.1%), 18.2% as neither easy nor difficult, 14.2% as difficult, and 1.5% did not know (Supplementary Table 1). Greater percentages of people without a medical cannabis prescription – both those who reported using medically and those who do not report medical use – reported perceiving access as more difficult than people with a medical prescription (43.7% and 40.1% vs 14.2%, respectively; *χ* ^2^ = 617.1, *p* < 0.001).

### Product use

**Figure 1.**
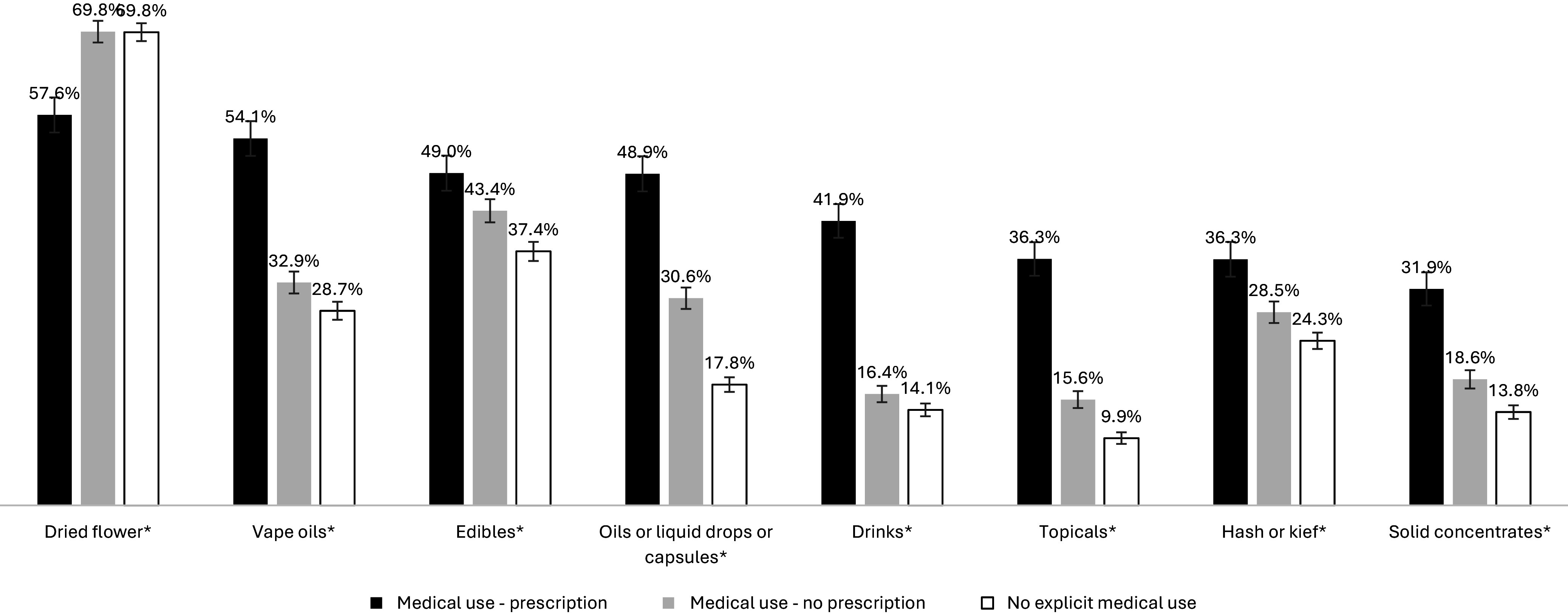
Any use of each product in the past year among respondents with a medical cannabis prescription, those without a prescription but using medically, and those without reporting any medical use (*n* = 4,415). *Note:* * indicates a statistically significant difference between groups at the 5% level using a univariable quasi-Poisson regression analysis with a log-link function. Dried flower [a,b]; Vape oils [a,b,c]; Edibles [a,c]; Oils or liquid drops or capsules [a,b,c]; Drinks [a,b,c]; Topicals [a,b,c]; Hash or kief [a,c]; Solid concentrates [a,b,c] where a is *p* < 0.05 between ‘medical use – prescription’ and ‘no medical use’, b is *p* < 0.05 between ‘medical use – prescription’ and ‘medical use – no prescription’ and c is *p* < 0.05 between ‘medical use – no prescription’ and ‘no medical use’. Figure 1. long description.

**Figure 2.**
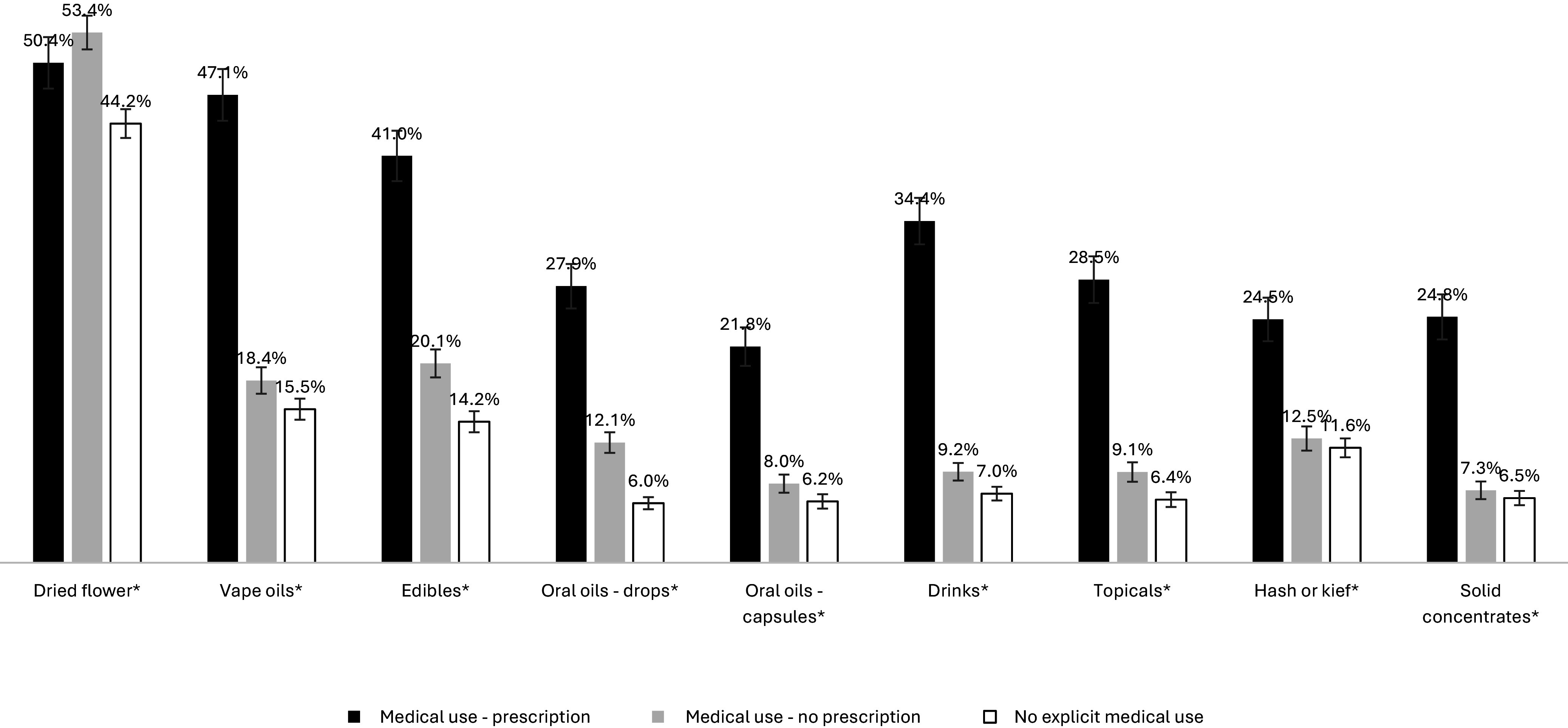
Monthly or more frequent use of each product in the past year among respondents with a medical cannabis prescription, those without a prescription but using medically, and those without reporting any medical use (*n* = 4,415). *Note*: * indicates a statistically significant difference between groups at the 5% level using a univariable quasi-Poisson regression analysis with a log-link function. Dried flower[c]; Vape oils[a,b]; Edibles[a,b,c]; Oils or liquid drops[a,b,c], oils or liquid capsules[a,b]; Drinks[a,b,c]; Topicals[a,b,c]; Hash or kief[a,b]; Solid concentrates[a,b] where a is *p* < 0.05 between ‘medical use – prescription’ and ‘no medical use’, b is *p* < 0.05 between ‘medical use – prescription’ and ‘medical use – no prescription’ and c is *p* < 0.05 between ‘medical use – no prescription’ and ‘no medical use’. Figure 2. long description.

People who reported receiving a medical cannabis prescription had greater percentages of past-year use than people without a prescription who reported medical use, and people who did not report medical use, for all products except dried flower (Figure 1). For dried flower, people who reported receiving a prescription had lower percentages of past-year use (all *p* < 0.05). People who reported receiving a medical cannabis prescription had greater percentages of MMF use in the past year than people without a prescription for medical cannabis but who reported using medically, for all products (all *p* < 0.05) except dried flower (*p* > 0.05) (Figure 2).

Tables 3 and 4 display regression analyses for MMF use. People who reported receiving a medical cannabis prescription had a higher probability of MMF use of oils or liquid drops (aRR = 3.51; 95% CI: 2.73–4.52), oil or liquid capsules (aRR = 2.63; 1.99–3.47), vape oils (aRR = 2.40; 2.03–2.84), edibles (aRR = 2.41; 2.01–2.90), cannabis drinks (aRR = 3.39; 2.69–4.26), solid concentrates (aRR = 3.10; 2.29–4.21), hash or kief (aRR = 1.82; 1.44–2.30), and topicals (aRR = 3.42; 2.58–4.51) than people not reporting medical use, after adjusting for covariates. People without a prescription who reported medical use had a higher probability of MMF use of dried flower (aRR = 1.18; 1.09–1.29), oils or liquid drops (aRR = 1.93; 1.50–2.49), vape oils (aRR = 1.27; 1.06–1.52), edibles (aRR = 1.49; 1.24–1.79), cannabis drinks (aRR = 1.44; 1.11–1.87), and topicals (aRR = 1.46; 1.09–1.97) than people not reporting medical use, after adjusting for covariates. Supplementary Tables 2 and 3 display results from the sensitivity analyses, which demonstrated a similar pattern of results.

**Table 3.** Weighted regression analyses on the monthly or more frequent use of cannabis products and medical cannabis prescriptions (*n* = 4,273) Table 3. long description.

	Monthly or more frequent use of (vs other)				
	Dried flower	Oil or liquid drops	Oil or liquid capsules	Vape oils	Edibles
	**aRR (95% CI)**				
**Medical cannabis use status** (vs no medical use)					
People with a prescription	1.09 (0.97, 1.23)	**3.51 (2.73, 4.52)**	**2.63 (1.99, 3.47)**	**2.40 (2.03, 2.84)**	**2.41 (2.01, 2.90)**
People without a prescription that use medically	**1.18 (1.09, 1.29)**	**1.93 (1.50, 2.49)**	1.29 (0.96, 1.74)	**1.27 (1.06, 1.52)**	**1.49 (1.24, 1.79)**
**Age** (vs 56–65)					
16–25	0.93 (0.80, 1.09)	0.87 (0.58, 1.33)	**2.59 (1.30, 5.16)**	**2.70 (1.74, 4.20)**	**2.29 (1.56, 3.37)**
26–35	1.13 (0.99, 1.31)	1.12 (0.77, 1.63)	**3.03 (1.59, 5.78)**	**2.48 (1.61, 3.82)**	**2.03 (1.39, 2.97)**
36–45	**1.16 (1.01, 1.33)**	1.38 (0.97, 1.97)	**3.11 (1.64, 5.89)**	**2.39 (1.55, 3.67)**	**2.08 (1.43, 3.03)**
46–55	1.07 (0.91, 1.26)	0.82 (0.53, 1.27)	**2.77 (1.37, 5.61)**	1.23 (0.75, 2.03)	1.08 (0.69, 1.70)
**Sex at birth** (vs Male)					
Female	**0.82 (0.76, 0.89)**	1.08 (0.89, 1.32)	**0.79 (0.62, 0.99)**	**0.74 (0.63, 0.85)**	0.87 (0.74, 1.01)
**Race/ethnicity** (vs White)					
Asian or Asian British	**0.68 (0.54, 0.86)**	0.99 (0.63, 1.59)	1.32 (0.80, 2.16)	**1.36 (1.05, 1.76)**	0.86 (0.60, 1.22)
Black, Black British, Caribbean or African	1.10 (0.97, 1.25)	1.09 (0.77, 1.54)	1.08 (0.77, 1.52)	0.93 (0.75, 1.15)	0.93 (0.75, 1.16)
Mixed or multiple ethnic groups	1.13 (0.95, 1.34)	0.99 (0.63, 1.59)	0.95 (0.56, 1.61)	0.84 (0.63, 1.12)	0.97 (0.72, 1.31)
Other/Unstated	0.89 (0.55, 1.39)	**0.13 (0.02, 0.92)**	**0.11 (0.02, 0.83)**	1.47 (0.84, 2.57)	1.17 (0.58, 2.36)
**Highest level of education** (vs less than high school)					
High school diploma	0.94 (0.82, 1.07)	**2.03 (1.23, 3.34)**	1.04 (0.56, 1.92)	**1.54 (1.08, 2.19)**	1.38 (0.97, 1.96)
Some college or technical vocation	**0.80 (0.71, 0.90)**	**1.91 (1.20, 3.02)**	1.30 (0.76, 2.21)	**1.47 (1.07, 2.00)**	1.26 (0.92, 1.73)
Bachelor’s degree or higher	**0.82 (0.73, 0.92)**	**2.06 (1.30, 3.26)**	1.40 (0.84, 2.34)	**1.50 (1.10, 2.05)**	**1.42 (1.04, 1.93)**
**Income adequacy** (vs very/difficult)					
Neither easy nor difficult	0.99 (0.90, 1.09)	0.88 (0.65, 1.19)	0.90 (0.63, 1.29)	1.21 (0.97, 1.51)	1.23 (0.97, 1.56)
Very Easy or easy	0.96 (0.87, 1.07)	1.12 (0.85, 1.46)	**1.39 (1.00, 1.93)**	**1.34 (1.10, 1.64)**	**1.62 (1.31, 2.00)**
Unstated	1.07 (0.81, 1.41)	0.24 (0.06, 1.00)	0.13 (0.02, 1.00)	0.95 (0.39, 2.29)	1.25 (0.65, 2.40)
**Region** (vs England no London)					
London	1.05 (0.96, 1.15)	0.99 (0.81, 1.21)	**1.33 (1.06, 1.67)**	**1.20 (1.04, 1.38)**	1.01 (0.87, 1.18)
Wales	1.14 (0.95, 1.36)	1.07 (0.64, 1.79)	1.34 (0.73, 2.45)	0.80 (0.45, 1.44)	**0.55 (0.35, 0.87)**
Scotland	**1.16 (1.02, 1.32)**	0.72 (0.48, 1.08)	1.19 (0.76, 1.85)	1.01 (0.76, 1.34)	1.02 (0.78, 1.34)
Northern Ireland	0.97 (0.73, 1.29)	0.87 (0.43, 1.79)	1.13 (0.51, 2.51)	1.55 (0.94, 2.55)	1.07 (0.64, 1.80)
**Year** (vs 2023)					
2024	1.01 (0.93, 1.10)	1.05 (0.84, 1.30)	1.10 (0.82, 1.46)	1.08 (0.92, 1.27)	0.95 (0.80, 1.13)
**Survey device used** (vs smartphone)					
Tablet	0.85 (0.66, 1.09)	1.03 (0.58, 1.84)	1.24 (0.63, 2.41)	1.33 (0.86, 2.06)	0.61 (0.32, 1.13)
Computer	0.92 (0.84, 1.01)	1.19 (0.96, 1.48)	1.32 (0.99, 1.74)	1.04 (0.89, 1.22)	0.91 (0.77, 1.09)

*Note:* Bolded values are statistically significant at the 5% level.

**Table 4.** Weighted regression analyses on the monthly or more frequent use of cannabis products and medical cannabis prescriptions (*n* = 4,273) Table 4. long description.

	Monthly or more frequent use of (vs Other)			
	Drinks	Solid Concentrates	Hash or kief	Topicals
	**aRR (95% CI)**			
**Medical cannabis use status** (vs no medical use)				
People with a prescription	**3.39 (2.69, 4.26)**	**3.10 (2.29, 4.21)**	**1.82 (1.44, 2.30)**	**3.42 (2.58, 4.51)**
People without a prescription that use medically	**1.44 (1.11, 1.87)**	1.19 (0.87, 1.63)	1.09 (0.86, 1.39)	**1.46 (1.09, 1.97)**
**Age** *(vs 56–65)*				
16–25	**3.88 (1.48, 10.19)**	**5.87 (2.19, 15.77)**	**1.70 (1.07, 2.70)**	**2.70 (1.43, 5.09)**
26–35	**4.83 (1.88, 12.44)**	**5.63 (2.15, 14.81)**	1.46 (0.92, 2.34)	**2.46 (1.33, 4.58)**
36–45	**4.74 (1.86, 12.05)**	**6.81 (2.61, 17.78)**	**2.16 (1.39, 3.34)**	**2.96 (1.62, 5.40)**
46–55	**2.86 (1.06, 7.69)**	**2.92 (1.04, 8.17)**	1.53 (0.92, 2.53)	1.48 (0.74, 2.94)
**Sex at birth** (vs Male)				
Female	**0.74 (0.61, 0.90)**	**0.77 (0.60, 0.99)**	**0.62 (0.50, 0.77)**	0.98 (0.79, 1.23)
**Race/ethnicity** (vs White)				
Asian or Asian British	1.15 (0.76, 1.75)	0.90 (0.54, 1.51)	1.12 (0.75, 1.66)	1.44 (0.92, 2.25)
Black, Black British, Caribbean or African	1.11 (0.86, 1.44)	0.97 (0.65, 1.46)	1.09 (0.76, 1.57)	1.12 (0.79, 1.59)
Mixed or multiple ethnic groups	0.86 (0.60, 1.25)	0.99 (0.60, 1.63)	1.21 (0.82, 1.78)	0.73 (0.48, 1.12)
Other/Unstated	0.48 (0.17, 1.35)	0.27 (0.06, 1.15)	1.85 (0.90, 3.79)	0.57 (0.16, 2.00)
**Highest level of education** (vs less than high school)				
High school diploma	1.70 (0.88, 3.30)	1.09 (0.62, 1.92)	1.33 (0.90, 1.96)	1.65 (0.87, 3.11)
Some college or technical vocation	**2.12 (1.19, 3.75)**	1.00 (0.61, 1.67)	1.25 (0.88, 1.78)	1.59 (0.89, 2.83)
Bachelor’s degree or higher	**3.15 (1.79, 5.54)**	1.00 (0.62, 1.61)	0.90 (0.63, 1.29)	**1.85 (1.06, 3.24)**
**Income adequacy** (vs very/difficult)				
Neither easy nor difficult	0.99 (0.72, 1.38)	1.03 (0.70, 1.52)	0.83 (0.63, 1.09)	1.16 (0.81, 1.67)
Very easy or easy	**1.46 (1.09, 1.97)**	1.18 (0.86, 1.62)	0.88 (0.68, 1.12)	**1.43 (1.03, 1.98)**
Unstated	0.70 (0.23, 2.15)	1.33 (0.46, 3.79)	0.47 (0.15, 1.42)	0.98 (0.24, 4.00)
**Region** (vs England no London*)*				
London	**1.43 (1.19, 1.73)**	**1.32 (1.05, 1.67)**	1.10 (0.89, 1.36)	1.20 (0.96, 1.50)
Wales	1.37 (0.81, 2.33)	0.63 (0.34, 1.18)	**0.40 (0.21, 0.74)**	1.13 (0.61, 2.07)
Scotland	0.75 (0.47, 1.20)	1.04 (0.62, 1.74)	1.13 (0.79, 1.63)	0.91 (0.58, 1.43)
Northern Ireland	1.12 (0.54, 2.32)	1.27 (0.42, 3.81)	0.94 (0.43, 2.06)	2.01 (0.95, 4.25)
**Year** (vs 2023)				
2024	0.99 (0.80, 1.23)	0.93 (0.70, 1.24)	0.94 (0.75, 1.17)	0.81 (0.63, 1.06)
**Survey device used** (vs smartphone)				
Tablet	1.14 (0.57, 2.28)	0.78 (0.26, 2.33)	0.65 (0.31, 1.35)	1.06 (0.49, 2.29)
Computer	0.96 (0.78, 1.17)	1.19 (0.92, 1.54)	1.01 (0.81, 1.25)	**1.37 (1.06, 1.76)**

*Note:* Bolded values are statistically significant at the 5% level.

### Characteristics of those who asked for and received a medical cannabis prescription

People who ever reported *asking* for a medical prescription (aRR = 2.54; 2.15–3.01) and people who reported *receiving* a medical prescription (aRR = 3.09; 2.51–3.79) in the past year had a higher probability of screening positive for high-risk use (Supplementary Table 4). People who had a higher probability of ever asking for a medical cannabis prescription and receiving a medical cannabis prescription in the past year were those aged 26–45, male-at-birth, educated to some college/technical vocation or higher, and residing in London.

## Discussion

To the best of our knowledge, this is the first study to examine the products and sources used among people with and without a medical cannabis prescription in the United Kingdom. The study has four primary findings: First, close to one in two people who used cannabis reported using cannabis for medical purposes, but only one in eight reported receiving a medical cannabis prescription in the past year. Second, there is a diversity of sources used to obtain cannabis; only one in ten people with a prescription obtained all their cannabis from a legal medical prescription, and a dealer was the most commonly used source. Third, people who reported receiving a medical cannabis prescription had a higher probability of consuming processed non-flower products monthly or more frequently than those without a prescription or those who did not report medical use. Fourth, people who ever asked for a medical prescription and people who reported having a medical prescription in the past year had a higher probability of screening positive for high-risk use.

Among those who reported using cannabis for medical purposes, a larger proportion reported using medically without a prescription than those who reported receiving a prescription in the past year. A similar disparity was demonstrated in a report by the Center for Medicinal Cannabis, which found that 1.4 million people in the United Kingdom were consuming cannabis for medical purposes, yet during the same period of the survey, fewer than 100 private prescriptions had been issued (Nutt, Bazire, Phillips, & Schlag, 2020). Another survey in 2022 concluded a higher figure – that 1.7 million people were consuming illegal cannabis to treat a health condition (Erridge et al., 2024). While our study suggests a larger proportion of people who self-reported cannabis use for medical purposes were accessing legal medical cannabis, there still appears to be an unmet demand for those self-medicating without access to formal medical guidance or oversight. However, it may be the case that some people reporting medical use are unlikely to be approved for a prescription if they do not have an adequate need, if practitioners have concerns about prescribing cannabis for their condition, or if all other treatments have not been exhausted, as per UK guidance. Indeed, a recent meta-analysis determined that while there was some low-quality evidence that cannabinoids reduced symptoms of some mental health and substance use disorders, there was no benefit of cannabinoids for the treatment of anxiety and an absence of data for the treatment of depression, two conditions for which people commonly report using cannabis for (Wilson et al., 2026). Furthermore, we found that the number reporting access to cannabis via NHS prescription was higher than expected, suggesting that some people may misunderstand the distinction between public and private prescriptions.

There is a diversity of sources used to obtain cannabis, even among those who reported receiving a prescription. Indeed, only one in ten people who reported having a medical cannabis prescription obtained all of their cannabis through a legal prescription. Most reported obtaining only *some* of their cannabis from a prescription, and the most common source used by those with a prescription was a dealer. It may be that those who reported having a prescription also consume cannabis for nonmedical purposes (i.e. recreationally) and used a different source for each purpose. Alternatively, they may experience problems with legal medical cannabis that do not meet consumer needs (e.g. based on availability, product, and/or price). In a qualitative study exploring the experiences of UK medical cannabis patients, participants reported prohibitive costs and supply issues, noting that prescriptions went out of stock or products were low quality (e.g. mold) (Wilson & McGrath, 2023). Moreover, in the current study, close to half of people without a prescription who self-disclosed medical use reported perceiving access to a prescription as difficult, perhaps due to health, financial, or competency barriers, or due to assumptions about whether the medical condition in question would be prescribed at all. This is consistent with a survey exploring why UK respondents who obtained their medical cannabis illegally did so rather than sourcing it legally; the most common reason cited was the presumed difficulties in access (Erridge et al., 2024). Our study demonstrates that there is a ‘mixed economy’ in terms of sourcing cannabis, whether for medical or recreational purposes. Indeed, healthcare professionals in contact with those with a medical cannabis prescription should be aware that the prescribed cannabis may not be the only cannabis people are consuming and should incorporate any additional consumption into their assessment and recommendations moving forward. Medical cannabis prescribers should be aware that people receiving prescribed cannabis may also be using additional cannabis outside of the formulation, dose, and treatment regimen that they prescribe. This could increase their cumulative THC exposure and risk of adverse events. Further research should investigate why people who use cannabis for medical purposes obtain it from outside the legal market, and what the illegal market offers that the legal medical market does not.

People who reported having a medical cannabis prescription had a higher probability of frequently consuming processed products, which typically contain higher potencies than dried flower, than those who do not report medical use. Only certain types of products are permitted in the legal medical market, and while one may expect to see more ‘medical’ products being consumed by those with a prescription (e.g. oils and capsules), those with a prescription are also frequently consuming products that are not permitted on the legal medical market (e.g. hash or solid concentrates). It is important to note that people with prescriptions may also be consuming cannabis for recreational purposes, with the other products potentially obtained illegally. Regardless, evidence points to an association between higher potency cannabis (and frequent use) with increased risk of cannabis use disorder (Craft et al., 2020; Freeman & Winstock, 2015; Petrilli et al., 2022). As estimates suggest cannabis use disorder affects one in four people who use medical cannabis (Dawson et al., 2024; Hsu et al., 2026); this is an important health outcome that should be discussed among people accessing medical cannabis. Indeed, in the current study, respondents who reported having a prescription had a higher probability of screening positive for high-risk use. Contact with healthcare professionals when accessing prescriptions for medical cannabis could consider whether the person who has or is requesting a prescription is at risk of high-risk use, as well as discuss the broader risks of cannabis use disorder. Moreover, contact with healthcare professionals could also use the opportunity to discuss with people who have or are requesting a prescription how the type of product used, frequency of use, and total THC exposure might influence risk of cannabis use disorder and other health outcomes. For example, safer use limits on THC consumption could be used to minimize the risk of cannabis use disorder (Lees Thorne et al., 2025).

## Limitations

This study is subject to limitations common to survey research. Respondents were recruited using non-probability-based sampling and are therefore not necessarily nationally representative. However, the data were weighted by age, sex, region, ethnicity, education, and cannabis use to align with national distributions.

Self-reported data are subject to social desirability biases. Cannabis is an illegal drug in the United Kingdom; therefore, patterns of cannabis use may be underreported or misrepresented. Indeed, while medical cannabis is legal in the United Kingdom, there is stigma surrounding its use for medical purposes (Troup, Erridge, Ciesluk, & Sodergren, 2022). However, the survey included a data integrity question, and those who reported not answering questions honestly were excluded. In addition, this survey was self-administered online, which, compared to interviewer-assisted surveys, can reduce social desirability biases by providing anonymity (Hays, Liu, & Kapteyn, 2015).

The current study used a clinical screening tool for high-risk cannabis use (CUDIT-SF) that is general to cannabis use and not tailored to recreational or medical cannabis use, nor specifically designed for self-reported surveys. However, a strength of the CUDIT-SF is that it was validated against a DSM-5 cannabis use disorder diagnosis in a sample of people recruited from a medical cannabis dispensary, as well as an independent sample of people reporting using cannabis for recreational purposes (Bonn-Miller et al., 2016). Previous research has also demonstrated that clinical interviewers capture similar rates of use to self-reported surveys (Gorfinkel, Stohl, Shmulewitz, & Hasin, 2024).

Finally, cannabis use for medical purposes was self-reported, and responses may reflect the specific measures used in the survey (Graham et al., 2025). Moreover, cannabis use for medical purposes, as reported by respondents, may not accurately reflect medical use or need as identified by a healthcare professional.

## Conclusions

More people use cannabis for medical purposes without a prescription than with one. Medical cannabis prescriptions in the United Kingdom may not be meeting the demands or needs of the people reporting medical use, whether with or without a prescription, as the illegal market continues to be utilized. Furthermore, people with a prescription use processed products more frequently than those without, and these typically contain higher potencies than dried flower. Contact with healthcare professionals when accessing prescriptions for medical cannabis use should discuss risks of higher potency products, frequency of use, and total THC exposure in relation to cannabis use disorder and other health outcomes.

## Supporting information

10.1017/S0033291726105327.sm001 Wadsworth et al. supplementary material Wadsworth et al. supplementary material

## Data Availability

The data that support the findings of this study are available from the International Cannabis Policy Study (www.cannabisproject.ca) but restrictions apply to the availability of these data and so are not publicly available. Data are however available from the authors upon reasonable request.

## Supplementary material

The supplementary material for this article can be found at http://doi.org/10.1017/S0033291726105327.

## Long descriptions

### Table 1. Long description

The table contains three columns: Characteristic, Unweighted percent (n), and Weighted percent (n).

Year: 2023 is 36.0 percent unweighted and 50.6 percent weighted. 2024 is 64.0 percent unweighted and 49.4 percent weighted.

Age: The largest weighted group is 16 to 25 at 37.6 percent, followed by 26 to 35 at 23.7 percent, 36 to 45 at 22.1 percent, 46 to 55 at 9.3 percent, and 56 to 65 at 7.3 percent.

Sex at birth: Weighted data shows 60.3 percent Male and 39.7 percent Female.

Race/ethnicity: White is the largest group at 80.3 percent weighted, followed by Black, Black British, Caribbean or African at 7.6 percent, Asian or Asian British at 5.5 percent, and Mixed or multiple ethnic groups at 5.1 percent.

Highest level of education: Weighted percentages are 19.2 percent Less than high school, 17.6 percent High school diploma, 34.0 percent Some college or technical vocation, and 26.8 percent Bachelor’s degree or higher.

Income adequacy: 37.9 percent weighted report Very Easy or easy, 32.9 percent report Neither easy nor difficult, and 26.6 percent report Very difficult or difficult.

Region: England not including London is the largest weighted region at 59.8 percent, followed by London at 21.8 percent, Scotland at 11.8 percent, Wales at 3.9 percent, and Northern Ireland at 2.7 percent.

Cannabis use frequency: Weighted data shows 28.4 percent use Daily or near daily, 18.8 percent Weekly, 24.7 percent Monthly, and 28.0 percent Less than monthly.

Medical cannabis status: 12.9 percent weighted have a prescription, 35.9 percent use medically without a prescription, and 51.2 percent report no medical use.

Navigate back to Table 1..

### Table 2. Long description

The table presents weighted percentages and unweighted counts for five main categories across four groups: Total past-year users (n = 4,415), People with a medical prescription (n = 676), People without a prescription using medically (n = 1,554), and People not reporting medical use (n = 2,125).

1. Ever asked for a medical prescription: 83.2% of those with a prescription said yes, compared to 13.1% of medical users without a prescription and 6.1% of non-medical users (p < 0.001).

2. Sourced cannabis from medical prescription in past year: 29.1% of the prescription group reported yes, while only 7.0% of medical users without a prescription and 2.1% of non-medical users did (p < 0.001).

3. Source of medical prescription: For the prescription group, 11.6% used an N H S prescription and 16.7% used a private prescription.

4. Amount of cannabis from legal prescription: In the prescription group, 62.9% reported ‘Some’ (1 to 99%) and 10.9% reported ‘All’ (100%). For other groups, ‘None’ was the most common response (41.0% and 40.5% respectively).

5. Sources of cannabis: ‘From a dealer (in person)' was the most common source across all groups, ranging from 47.2% to 54.1%. The prescription group had significantly higher rates of sourcing from a store or dispensary (36.3%) and growing their own (32.0%) compared to other groups.

6. High-risk use (C U D I T - S F score): The prescription group had the highest positive screen rate at 75.8%, compared to 45.5% for medical users without a prescription and 39.6% for non-medical users (p < 0.001).

Navigate back to Table 2..

### Figure 1. Long description

The x-axis lists eight cannabis product categories, each with three bars representing different user groups. The legend at the bottom identifies the bars: black for Medical use - prescription, light gray for Medical use - no prescription, and white for No explicit medical use. All categories are marked with an asterisk indicating statistical significance.

From left to right, the data percentages are:

* Dried flower: 57.6 percent, 69.8 percent, and 69.8 percent.

* Vape oils: 54.1 percent, 32.9 percent, and 28.7 percent.

* Edibles: 49.0 percent, 43.4 percent, and 37.4 percent.

* Oils or liquid drops or capsules: 48.9 percent, 30.6 percent, and 17.8 percent.

* Drinks: 41.9 percent, 16.4 percent, and 14.1 percent.

* Topicals: 36.3 percent, 15.6 percent, and 9.9 percent.

* Hash or kief: 36.3 percent, 28.5 percent, and 24.3 percent.

* Solid concentrates: 31.9 percent, 18.6 percent, and 13.8 percent.

Error bars are present on all columns. Except for dried flower, the Medical use - prescription group consistently shows the highest usage rates across all product types.

Navigate back to Figure 1..

### Figure 2. Long description

The x-axis lists nine cannabis product categories, each with three bars representing Medical use - prescription (black), Medical use - no prescription (light gray), and No explicit medical use (white outline). All categories are marked with an asterisk indicating statistical significance.

* Dried flower: Prescription 50.4 percent, No prescription 53.4 percent, No medical use 44.2 percent.

* Vape oils: Prescription 47.1 percent, No prescription 18.4 percent, No medical use 15.5 percent.

* Edibles: Prescription 41.0 percent, No prescription 20.1 percent, No medical use 14.2 percent.

* Oral oils - drops: Prescription 27.9 percent, No prescription 12.1 percent, No medical use 6.0 percent.

* Oral oils - capsules: Prescription 21.8 percent, No prescription 8.0 percent, No medical use 6.2 percent.

* Drinks: Prescription 34.4 percent, No prescription 9.2 percent, No medical use 7.0 percent.

* Topicals: Prescription 28.5 percent, No prescription 9.1 percent, No medical use 6.4 percent.

* Hash or kief: Prescription 24.5 percent, No prescription 12.5 percent, No medical use 11.6 percent.

* Solid concentrates: Prescription 24.8 percent, No prescription 7.3 percent, No medical use 6.5 percent.

Error bars are present on all columns, showing the highest usage rates among the prescription group for every category except dried flower.

Navigate back to Figure 2..

### Table 3. Long description

The table presents A R R values for monthly or more frequent use of five cannabis products: Dried flower, Oil or liquid drops, Oil or liquid capsules, Vape oils, and Edibles.

* Medical cannabis use status: Compared to no medical use, people with a prescription show significantly higher use of oil drops (3.51), capsules (2.63), vape oils (2.40), and edibles (2.41). People without a prescription using medically show a significant increase across all products except capsules.

* Age: Compared to ages 56 to 65, younger groups (16 to 45) show significantly higher use of capsules, vape oils, and edibles, with a R R values ranging from 2.03 to 3.11.

* Sex at birth: Females show significantly lower use of dried flower (0.82), capsules (0.79), and vape oils (0.74) compared to males.

* Race/ethnicity: Compared to White participants, Asian or Asian British participants show lower dried flower use (0.68) but higher vape oil use (1.36).

* Education: Compared to less than high school, those with a Bachelor's degree or higher show significantly higher use of oil drops (2.06), vape oils (1.50), and edibles (1.42), but lower use of dried flower (0.82).

* Income adequacy: Those finding it very easy or easy show higher use of capsules (1.39), vape oils (1.34), and edibles (1.62) compared to those finding it difficult.

* Region: Compared to England (excluding London), London residents show higher use of capsules (1.33) and vape oils (1.20), while Scotland shows higher dried flower use (1.16) and Wales shows lower edible use (0.55).

Navigate back to Table 3..

### Table 4. Long description

The table presents adjusted Relative Risk (A R R) with 95 percent Confidence Intervals (C I) for the monthly use of Drinks, Solid Concentrates, Hash or kief, and Topicals.

* Medical cannabis use status: Compared to no medical use, people with a prescription show significantly higher risk across all products: Drinks (3.39), Solid Concentrates (3.10), Hash or kief (1.82), and Topicals (3.42). People without a prescription using medically show significant risk for Drinks (1.44) and Topicals (1.46).

* Age: Compared to ages 56 to 65, all younger age groups (16 to 25, 26 to 35, 36 to 45) show significantly higher risk for most products, with the highest risk in Solid Concentrates (ranging from 5.63 to 6.81).

* Sex at birth: Females show significantly lower risk than males for Drinks (0.74), Solid Concentrates (0.77), and Hash or kief (0.62).

* Race/ethnicity: No significant differences were found across Asian, Black, or Mixed groups compared to White respondents.

* Education: Compared to less than high school, those with a Bachelor’s degree or higher have significantly higher risk for Drinks (3.15) and Topicals (1.85).

* Income adequacy: Those finding it ‘Very easy or easy’ have higher risk for Drinks (1.46) and Topicals (1.43) compared to those finding it difficult.

* Region: Compared to England (excluding London), London residents have higher risk for Drinks (1.43) and Solid Concentrates (1.32), while Wales has lower risk for Hash or kief (0.40).

* Survey device: Computer users show higher risk for Topicals (1.37) compared to smartphone users.

Navigate back to Table 4..
